# Quantitative proteomic analysis after neuroprotective MyD88 inhibition in the retinal degeneration 10 mouse

**DOI:** 10.1111/jcmm.16893

**Published:** 2021-09-25

**Authors:** Tal Carmy‐Bennun, Ciara Myer, Sanjoy K. Bhattacharya, Abigail S. Hackam

**Affiliations:** ^1^ Bascom Palmer Eye Institute University of Miami Miller School of Medicine Miami FL USA; ^2^ Miami Integrative Metabolomics Research Center Miami FL USA

**Keywords:** crystallin, iTRAQ, MyD88, photoreceptors, proteomics, retinal degeneration, Toll‐like receptors

## Abstract

Progressive photoreceptor death occurs in blinding diseases such as retinitis pigmentosa. Myeloid differentiation primary response protein 88 (MyD88) is a central adaptor protein for innate immune system Toll‐like receptors (TLR) and induces cytokine secretion during retinal disease. We recently demonstrated that inhibiting MyD88 in mouse models of retinal degeneration led to increased photoreceptor survival, which was associated with altered cytokines and increased neuroprotective microglia. However, the identity of additional molecular changes associated with MyD88 inhibitor‐induced neuroprotection is not known. In this study, we used isobaric tags for relative and absolute quantification (iTRAQ) labelling followed by LC‐MS/MS for quantitative proteomic analysis on the *rd10* mouse model of retinal degeneration to identify protein pathways changed by MyD88 inhibition. Quantitative proteomics using iTRAQ LC‐MS/MS is a high‐throughput method ideal for providing insight into molecular pathways during disease and experimental treatments. Forty‐two proteins were differentially expressed in retinas from mice treated with MyD88 inhibitor compared with control. Notably, increased expression of multiple crystallins and chaperones that respond to cellular stress and have anti‐apoptotic properties was identified in the MyD88‐inhibited mice. These data suggest that inhibiting MyD88 enhances chaperone‐mediated retinal protection pathways. Therefore, this study provides insight into molecular events contributing to photoreceptor protection from modulating inflammation.

## 1 INTRODUCTION

Photoreceptors are light‐sensing neurons in the retina that are essential for vision. In many types of ocular disease, including common degenerative diseases such as age‐related macular degeneration and retinitis pigmentosa, dysfunction or death of photoreceptors leads to reduced vision and eventual blindness. There is currently no treatment to prevent photoreceptor death. Because inflammation plays a role in retinal degenerations, there is a great interest in the retina field in developing inflammatory modulators as therapeutic targets. Toll‐like receptors (TLR) are a large family of innate immune system receptors that respond to specific pathogen‐associated molecular patterns and lead to immune cell activation and induction of a wide variety of inflammatory responses.^1^ Our group and others have demonstrated that TLRs contribute to neuronal death in inherited and induced retinal degenerations.^2^, ^3^, ^4^, ^5^ The Myeloid differentiation primary response protein 88 (MyD88) protein is a central adaptor molecule for most TLRs and mediates TLR‐induced signalling in inflammatory and non‐inflammatory cells. Therefore, MyD88 has been considered as a potential target to block aberrant activation of TLR during neurodegeneration.^5^, ^6^, ^7^, ^8^, ^9^, ^10^, ^11^, ^12^, ^13^

We recently demonstrated that inhibiting MyD88 by gene knockout^14^ and pharmacologic inhibitor^2^ led to higher photoreceptor survival in two mouse models of retinal degeneration. Furthermore, neuroprotection from inhibiting MyD88 in the *rd10* mouse model of retinal degeneration was associated with increased microglia/macrophage expressing the Arg1 neuroprotective marker and altered levels of several anti‐inflammatory cytokines.^2^ However, additional molecular changes associated with MyD88 inhibition are currently unknown. In this study, we were interested in identifying early molecular events after MyD88 inhibition as an important first step to understanding how it induces photoreceptor protection.

High‐performance liquid chromatography tandem mass spectrometry combined with isobaric tag labelling with iTRAQ is a sensitive, highly accurate and high‐throughput method to identify differentially expressed proteins. The iTRAQ method has not been reported for the analysis of the retinal proteome in photoreceptor degeneration, although it has been used to profile protein changes after retinal detachment,^15^ myopia^16^ and optic nerve transection.^17^ In this study, we performed iTRAQ quantitative proteomic analysis on *rd10* mouse retinas to characterize protein changes that may contribute to the protective effects of inhibiting MyD88. We identified altered expression of 42 proteins, including anti‐apoptotic crystallins, chaperones and regulators of protein biosynthesis. These findings suggest a link between modulating inflammatory pathways and chaperone expression and suggest that inhibiting MyD88‐mediated signalling may enhance intrinsic tissue‐protective pathways. Therefore, this study provides new insight into molecular changes that may contribute to photoreceptor protection and provide a foundation for understanding molecular mechanisms contributing to retinal homeostasis in *rd10* mice.

## 2 MATERIALS AND METHODS

### 2.1 Animal studies

All experiments using mice were approved by the Animal Care and Use Committee at the University of Miami and were performed in accordance with the ARVO statement for the Use of Animals in Ophthalmic and Vision research. The *retinal degeneration 10* mouse strain (*rd10*, B6.CXB1‐Pde6brd10/J) was purchased from Jackson Laboratory. The *rd10* line is homozygous for a mutation in the rod‐specific visual transduction *Pde6b* gene and is commonly used to model retinitis pigmentosa and test experimental therapies.^18^, ^19^, ^20^ The retinal degeneration phenotype in *rd10* is specific to the retina due to the causative gene, *Pde6b*, being exclusively expressed in rod photoreceptors. Mice of both sexes were used, and they were housed under 12‐h light‐dark cycle with ad libitum access to food and water. Littermates were used for control and inhibitor injections, and all animals were housed at an equivalent distance from the overhead light.

Mice at age post‐natal day 18 were intraperitoneally (IP) injected with MyD88 inhibitor peptide (IMG2005, Novus Biologicals) following the procedures of our recent study.^2^ The MyD88 inhibitor peptide contains a MyD88 homodimerization sequence (underlined, DRQIKIWFQNRRMKWKKRDVLPGT) and a protein transduction (PTD) sequence derived from antennapedia for cellular permeability. The control peptide contains only the PTD sequence. Both peptides were solubilized in sterile PBS according to the manufacturer's directions, aliquoted and stored frozen, then freshly diluted immediately prior to use. Mice (*n* = 4 MyD88 inhibitor peptide, *n* = 4 control peptide) were injected with MyD88 inhibitor peptide or control peptide at a concentration of 2 mg/kg body weight, which is the dose that induced maximum photoreceptor rescue in our previous study.^2^ The animals were sacrificed 3 days after injection to collect tissue for proteomics analysis. After enucleation, the lens was removed, and the retina was carefully separated from the eye cup. Investigators were masked to the identity of the injected compound for all analyses.

### 2.2 Protein extraction and sample preparation for ITRAQ analysis

Isolated retinas were homogenized in 300 µl of T‐PER buffer (Thermo Scientific # 78510) and then centrifuged at 1000 *g* for 10 min. The supernatant was collected in a separate tube, four volumes of cold acetone were added, and the samples were incubated overnight at −20°C. The samples were centrifuged at 10,000 *g* for 10 min and dried in a speed vacuum concentrator, then reconstituted in 0.5 M triethylammonium bicarbonate (TEAB). Protein concentrations were determined using a Bicinchoninic assay (BCA) (Thermo Fisher Scientific) according to the manufacturer's directions. The volume to obtain 40 µg of each sample was calculated, placed in a separate Eppendorf tube and dried in a speed vacuum concentrator. Each sample was reconstituted in 30 µl of 0.5 M TEAB. The proteins were denatured and reduced by adding 1 µl of 2% SDS and 1 µl of 110 mM tris‐(2‐carboxyethyl) phosphine (TCEP). The samples were vortexed, centrifuged at 12,000 x *g* for 5 min, incubated for 1 h at 60°C and then centrifuged again at 12,000 x *g* for 5 min. Proteins were alkylated by adding 1 µl of 84 mM iodoacetamide, vortexed and then centrifuged as in previous step. The samples were incubated at room temperature for 30 min in the dark then centrifuged at 12,000 x *g* for 5 min. Proteins were digested using sequence‐grade trypsin and incubated at 37°C overnight. ITRAQ Reagents (8‐plex) (Sciex #4390733) were added to samples and incubated at room temperature for 2 h, following our previous study.^21^ The reaction was quenched by adding 100 µl of LC‐MS grade water and incubated for another 30 min. All samples were combined into one tube, dried in a speed vacuum concentrator, washed three times with 100 µl of LC‐MS grade water and then stored dry at 20°C until analysis by high‐performance liquid chromatography tandem mass spectrometry (LC‐MS/MS). The ITRAQ sample was reconstituted in 30 µl of 2% acetonitrile in water with 0.1% formic acid prior to LC‐MS/MS analysis.

### 2.3 High‐performance liquid chromatography‐mass spectrometry

The trypsin‐digested proteins were analysed using a Q Exactive mass spectrometer and an Easy‐nLC 1000 equipped with a Nanospray Flex ion source (Thermo Fisher Scientific) operating in positive ion mode. The peptides were separated using an Acclaim PepMap RSLC 75 µm × 15 cm column (Thermo Fisher Scientific) with a flow rate of 450 nl/min. Solvent A was LC‐MS grade water with 0.1% formic acid, and solvent B was LC‐MS grade acetonitrile with 0.1% formic acid. The gradient ran from 2% solvent B to 30% solvent B over 57 min then to 80% solvent B over 6 min and was held at 80% solvent B for 12 min. The parameters of the ionization source were as follows: spray voltage was 1.8 kV, the capillary temperature was 250°C, the S‐lens radio frequency (RF) level was 50, and all gases were set to 0. The full scan mass spectrometry settings included a resolution of 70,000, maximum injection time of 100 ms, and an Automatic Gain Control (AGC) target of 1e6. The data‐dependent MS2 settings were set at a resolution of 17,500, injection time of 50 ms, AGC target of 2e5. The isolation window was 2 m/z, and the normalized collision energy was set to 28.

### 2.4 Protein identification and quantification in proteome Discoverer 2.2

Proteins were identified and quantified from LC‐MS/MS spectral data using Proteome Discoverer 2.2 software (Thermo Fisher Scientific). Raw files were searched against the *Mus musculus* entries in the Uniprot sequence database using the Sequest HT search engine. The following dynamic modifications were searched for +15.995 Daltons (Da) on methionine residues for oxidation, +304.205 Da on lysine residues for ITRAQ 8‐Plex, +304.205 Da on the N‐terminus of peptides for ITRAQ 8‐Plex and +42.011 Da on the N‐terminus of proteins for an acetyl group. Static modifications included +52.021 Da on cysteine residues for a carbamidomethyl group. The search parameters allowed for two missed trypsin cleavages. The precursor mass tolerance was 10 ppm, and the fragment mass tolerance was 0.02 Da. The percolator PSM validator was used with a maximum ΔCn of 0.05, target strict false discovery rate (FDR) of 0.01 and target relaxed FDR of 0.05. The validation was based on the q‐value. High confidence peptides were selected, and abundances were calculated for each isobaric tag.

### 2.5 Bioinformatics

The differentially expressed proteins were categorized with Gene ontology (GO) terms using http://geneontology.org/ with PANTHER Version 15.0 (released 2020‐02‐14)^22^ for enrichment analysis, using Fisher's exact test and *Mus musculus* as the reference list. Proteins with a *p*‐value < 0.05 were considered significantly enriched. Additionally, protein‐protein interaction networks using physical and functional interactions were analysed using the STRING database (Search Tool for the Retrieval of Interacting Genes/Proteins) v.11 (http://string‐db.org/)^23^ with a minimum interaction score of 0.7 for high confidence.

### 2.6 Statistical analysis

False discovery rate and enrichment analyses of the proteomics data and bioinformatics were performed by embedded software in the respective programmes.

### 2.7 Data sharing

The proteomics raw data have been deposited in the PRIDE (PRoteomics IDEntifications) database, Project accession: PXD024501, Project 10.6019/PXD024501.

## 3 RESULTS

### 3.1 Identification of differentially expressed proteins

To identify proteins that may stimulate neuroprotective pathways in *rd10* mice, and to capture initial changes that result from MyD88 inhibition, we used an early timepoint of 3 days after MyD88 inhibition. This timepoint is prior to significant photoreceptor death.^19^, ^20^, ^24^ We also reasoned that later timepoints would mostly reveal altered photoreceptor proteins, overwhelming the samples with degeneration‐associated proteins and would be less likely to reveal early mechanistic changes leading to increased photoreceptor survival.

A quantitative proteomics analysis was performed on retinas obtained from mice treated with MyD88 inhibitor peptide or control peptide. To identify differentially expressed proteins, highly sensitive combination iTRAQ LC‐MS/MS analysis was performed on total cellular proteins extracted from neural retina tissue. This analysis detected 5586 proteins in the mouse retinas (*n* = 4 MyD88 inhibitor peptide, *n* = 4 control peptide), of which 394 proteins were identified with high confidence (FDR *p* < 0.01) and 54 were medium confidence (FDR *p* < 0.05), for a total of 448 combined high and medium confidence proteins. The remaining 5138 proteins were detected at low confidence and were not analysed further. Between 1 to 26 unique peptides mapped to each high and medium confidence protein, 301 (out of 394) high confidence proteins had ≥2 unique matching peptides and 31 (out of 54) medium confidence proteins had ≥2 unique matching peptides. Furthermore, there was an average of 14% protein coverage for the high confidence proteins and average of 7% for the medium confidence proteins (Table S1).

The differentially expressed proteins were identified from the 448 high and medium confidence total proteins by selecting iTRAQ ratios of MyD88 inhibitor injected to control peptide injected mice that had fold changes >1.2 or <0.83 and with *p* < 0.05, as used in previous studies.^15^, ^25^ In total, 42 retinal proteins were differentially expressed: 20 proteins were upregulated in the MyD88 inhibitor injected mice and 22 proteins were downregulated (Table 1). The fold changes are relatively modest with a maximum change of 2.7‐fold, which is expected at this early timepoint prior to significant photoreceptor death.^19^, ^20^ Fifty‐seven of the high confidence proteins and 15 medium confidence proteins were not labelled with the isobaric tag and relative quantification and their relative expression could not be determined.

**TABLE 1:** Differentially expressed genes (20 upregulated, 22 downregulated) were identified in the 448 high and medium confidence total proteins using iTRAQ ratios of MyD88 inhibitor injected to control peptide injected that were >1.2 or <0.83

Uniprot accession	Description	Gene	Fold change (inhibitor/control)
Upregulated			
P62141	Ser/Thre‐protein phosphatase PP1‐beta	Ppp1cb	2.7274
Q9QXC6	Beta‐A3/A1 crystallin protein	Cryba1	2.452309
J3QJW3	Calcium‐dependent secretion activator 1	Cadps	2.432003
Q569M7	Cryaa protein	Cryaa	2.076538
Q4FZE6	40S ribosomal protein S7	Rps7	1.804698
Q3TEA8	Heterochromatin protein 1‐binding protein 3	Hp1bp3	1.640806
O35486	Gamma‐crystallin S	Crygs	1.587322
P46664	Adenylosuccinate synthetase isozyme 2	Adss	1.4961
A2RTH4	Crystallin, gamma E	Cryge	1.393776
Q3TVV6	Heterogeneous nuclear ribonucleoprotein U	Hnrnpu	1.349486
P23927	Alpha‐crystallin B chain	Cryab	1.346436
F6W687	Non‐histone chromosomal protein HMG‐17	Hmgn2	1.280787
Q9JJU9	Beta‐crystallin B3	Crybb3	1.2567
Q8VED9	Galectin‐related protein	Lgalsl	1.241524
P62702	40S ribosomal protein S4, X isoform	Rps4x	1.238388
D3YTR0	Adenomatous polyposis coli protein 2	Apc2	1.234013
A3RLD5	Gamma‐crystallin C	Crygc	1.228755
P11983	T‐complex protein 1 subunit alpha	Tcp1	1.228412
Q4KL76	Heat shock protein 1 (Chaperonin 10)	Hspe1	1.21914
Q3TCH2	Ubiquitin carboxyl‐terminal hydrolase	Uchl1	1.209669
Downregulated			
Q8CF71	Alpha‐actin‐2	Acta2	0.833601
G5E829	Plasma membrane calcium‐transporting ATPase 1	Atp2b1	0.829636
Q3TLX1	Nicotinamide phosphoribosyltransferase	Nampt	0.827507
Q80ZI9	WD repeat domain 1	Wdr1	0.82357
P10630	Eukaryotic initiation factor 4A‐II	Eif4a2	0.818182
A0A023J5Z7	ATP synthase subunit a	ATP6	0.813421
B4DE70	highly similar to syntaxin 3, variant C	Stx3	0.812188
Q9JKC6	Cell cycle exit and neuronal differentiation protein 1	Cend1	0.807501
Q3TQ70	Beta1 subunit of GTP‐binding protein	Gnb1	0.79051
Q3UE92	X‐prolyl aminopeptidase P1	Xpnpep1	0.784965
P15409	Rhodopsin	Rho	0.783501
Q6ZQ61	Matrin‐3	Matr3	0.781563
Q9CZD3	Glycine‐tRNA ligase	Gars	0.780325
Q9DAR7	m7GpppX diphosphatase	Dcps	0.778964
Q9D6F9	Tubulin beta‐4A	Tubb4a	0.774845
Q9WV34	MAGUK p55 subfamily member 2	Mpp2	0.764446
Q543U3	Amino acid transporter	Slc1a3	0.761894
P18572	Basigin	Bsg	0.7458
Q11011	Puromycin‐sensitive aminopeptidase	Npepps	0.745201
Q6ZWN5	40S ribosomal protein S9	Rps9	0.705544
P09405	Nucleolin	Ncl	0.687052
O54984	ATPase GET3	Asna1	0.59596

### 3.2 Functional categorization of differentially expressed proteins

Gene ontology analysis using PANTHER^22^ was used to categorize the 42 differentially expressed proteins, into “Biological process,” “Molecular function” and “Cellular component” categories using *Mus musculus* as the reference organism. The significance of the different GO terms in our dataset was determined using an enrichment analysis. The top four terms for enriched biological processes with the number of genes in each category included multicellular organism development, eye development, peptide metabolic process and response to hypoxia (Table 2). The top enriched molecular functions were eye lens structural constituents, unfolded protein binding, ligase activity and small molecule binding. There were no significantly enriched cellular components categories.

**TABLE 2:** GO functional annotation enrichment analysis of the 42 differentially expressed genes

	# Genes	Expected	Fold enrichment	*p*‐value	FDR
GO biological process					
Multicellular organismal process	28	14.44	1.94	3.41E‐05	3.84E‐02
Multicellular organism development	23	9.42	2.44	6.92E‐06	1.09E‐02
System development	22	8.21	2.68	2.66E‐06	4.66E‐03
Eye development	10	0.76	13.14	3.92E‐09	2.06E‐05
Organonitrogen compound biosynthetic process	10	2.13	4.7	3.95E‐05	3.89E‐02
Cellular amide metabolic process	8	1.34	5.96	5.16E‐05	4.29E‐02
Peptide metabolic process	7	0.88	7.94	2.72E‐05	3.58E‐02
Response to hypoxia	5	0.38	13.04	4.40E‐05	4.08E‐02
Nucleotide biosynthetic process	5	0.34	14.8	2.44E‐05	3.51E‐02
GO molecular function					
Structural constituent of eye lens	7	0.05	>100	2.28E‐13	1.06E‐09
Structural molecules activity	12	1.13	10.58	9.45E‐10	2.20E‐06
Unfolded protein binding	4	0.17	23.81	2.84E‐05	2.64E‐02
Ligase activity	5	0.32	15.52	1.95E‐05	2.27E‐02
Small molecule binding	16	4.82	3.32	8.91E‐06	1.38E‐05

Note

The number of genes, fold enrichment and FDR value are indicated for the two GO categories, biological process and molecular function.

**FIGURE 1:**
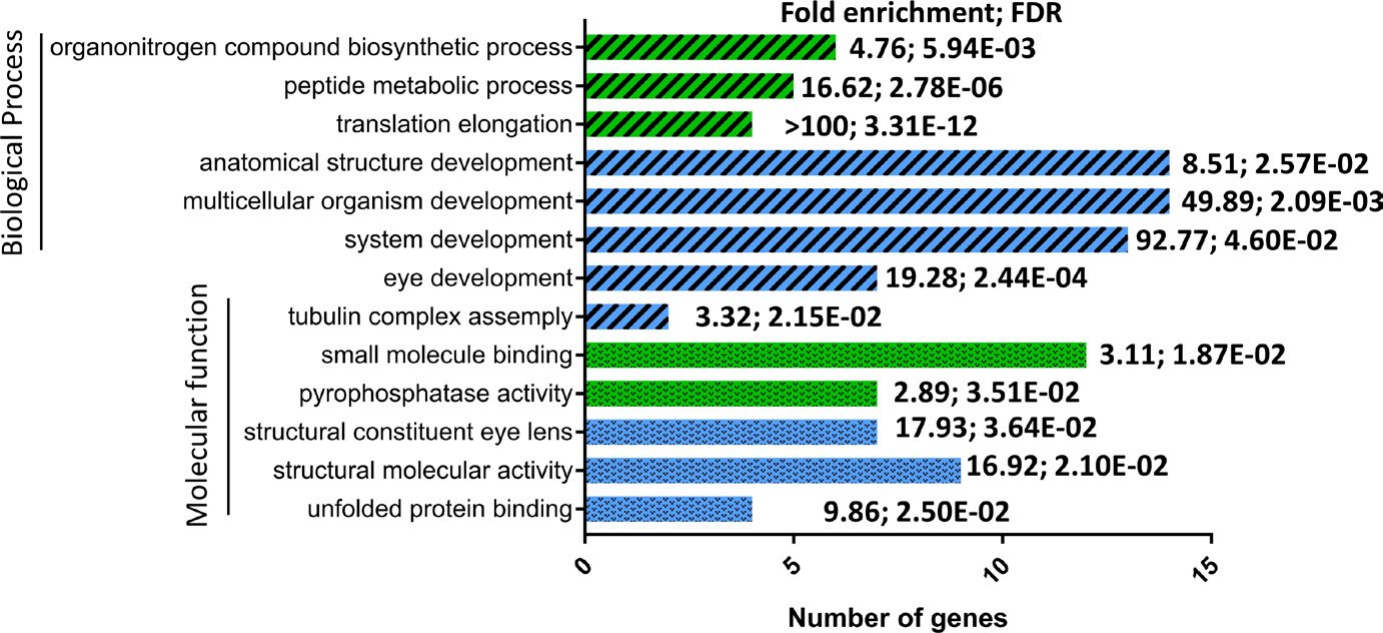
Gene ontology (GO) functional annotation enrichment analysis of the upregulated (blue) and downregulated (green) proteins. GO term categories of biological processes are indicated by cross‐hatching and molecular function by dots. The number of genes in each category is indicated on the *x*‐axis. The fold enrichment and false discovery rate (FDR) are indicated for each category (Fisher's exact test)

Enrichment analysis was also performed separately for the increased and decreased proteins. The 20 increased genes were significantly enriched in three molecular functions (structural constituent of eye lens, unfolded protein binding and structural molecular activity) and 13 biological processes (five distinct processes when redundancies were removed) (Figure 1). There were no significantly enriched cellular compartments for the increased proteins. In contrast, the 22 decreased proteins were categorized into two significantly enriched molecular functions (pyrophosphatase activity, small molecule binding), three distinct significantly enriched biological processes (peptide biosynthesis, organonitrogen compound biosynthesis and amide metabolic process) and three distinct significantly enriched cellular compartments (photoreceptor inner segment, outer segment cytoplasm) (Figure 1).

Interestingly, the upregulated proteins were enriched for crystallin genes. Out of 20 upregulated proteins, seven of them belong to the crystallin family (Table 1). Numerous studies have reported expression of alpha, beta and gamma crystallin proteins outside the lens within the photoreceptor, inner nuclear and ganglion cell layers of the retina.^26^, ^27^, ^28^ It is now understood that α‐crystallins belong to the small heat shock protein superfamily and play neuroprotective and stress response roles, whereas β‐ and γ‐crystallins are stress response and structural proteins.^27^ However, to exclude the possibility that the retina samples were contaminated with lens tissue, we searched the total 5586 identified proteins for lens‐specific and lens‐enriched proteins.^26^ The lens proteins GJA3, GJA8, GJA1, aquaporin 0, radixin and BFSP1 were not detected or were detected with only low confidence, and 10 crystallins that are the major structural components of lens were also not detected in the retina samples: CryβA2, CryβA4, CryβB2, CryγA, CryγB, CryγD, CryγF and CryγN. Therefore, the absence of these lens proteins indicates that the detection of known extra‐lenticular crystallins was not likely due to contamination of the retina tissue with lens during the tissue dissociation steps.

### 3.3 Protein‐protein interaction network analysis

**FIGURE 2:**
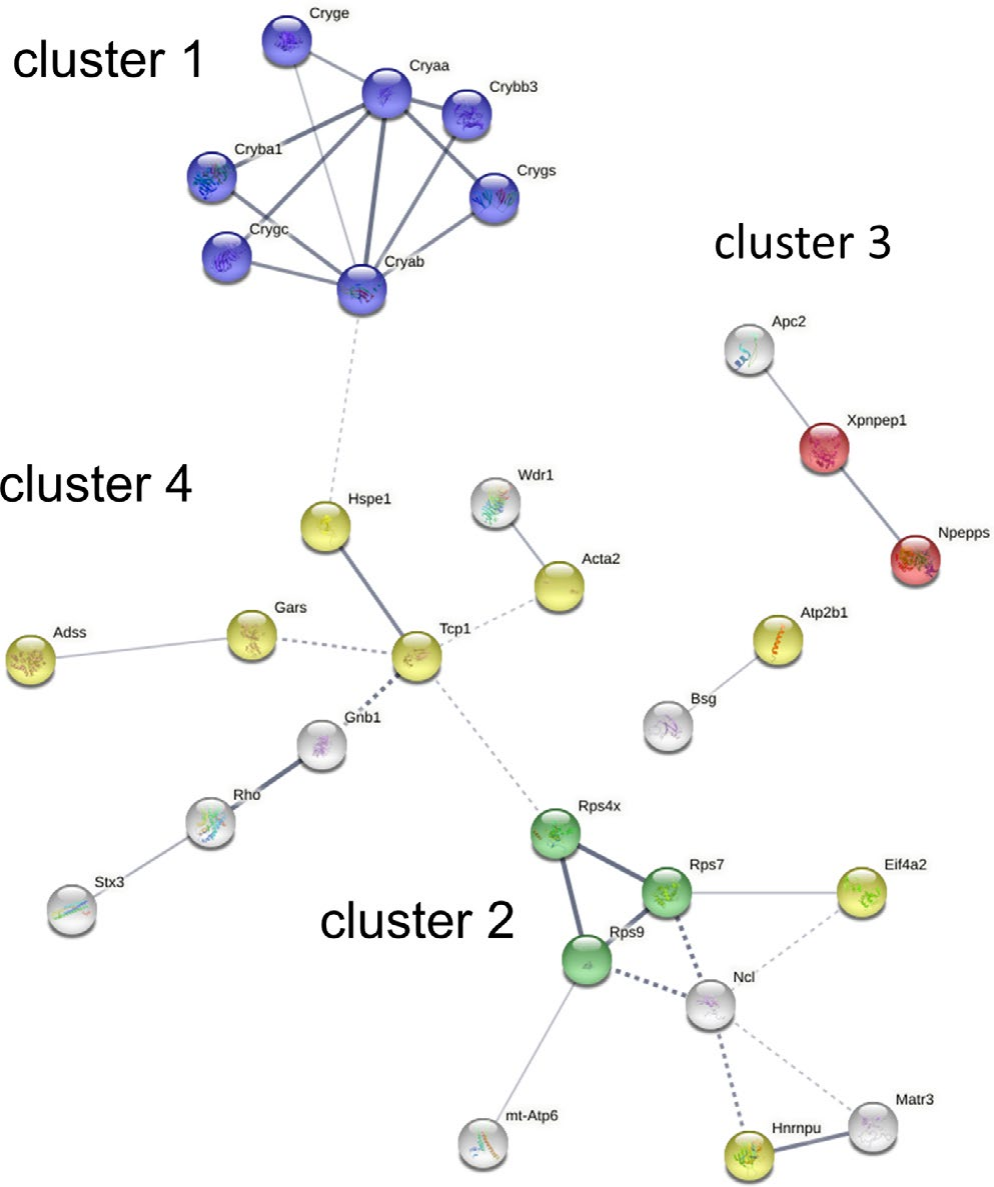
Protein‐protein interaction network analysis. STRING analysis using high confidence level identified three enriched protein‐protein interaction networks in the 42 differentially expressed proteins. Connected functional and/or physical interacting proteins are represented by nodes, and the interaction between two proteins is represented by lines. The thickness of the lines indicates the strength of the data supporting the interaction; the dotted line indicates low edge confidence. Notable functional clusters are indicated and colour‐coded as follows: lens proteins/apoptotic process (cluster 1, blue), cytosolic small ribosome subunit (cluster 2, green), aminopeptidase activity (cluster 3, red) and purine ribonucleotide binding (cluster 4, yellow)

To explore potential signalling pathways among the differentially expressed proteins, protein‐protein interaction networks were investigated in silico using STRING v11 analysis.^23^ Several clusters representing enriched functional and physical associations were identified in the 42 differentially expressed proteins (Figure 2). At a high confidence level, the differentially expressed proteins were grouped into several independent interaction networks, with 35 edges, average node degree of 1.67 and a local clustering coefficient of 0.429. The expected number of edges of 16 and the PPI enrichment *p*‐value was 1.48e‐05 indicating that the network has significantly more interactions than expected compared with a random similarly sized group of proteins, suggesting that the proteins are likely connected in a biological pathway or function. Four clusters are noted: two highly connected clusters representing lens protein/apoptotic processes (cluster 1: crystallins CryαA, CryαB, CryγS, CryβA1, CryγE, CryγC, CryβB3) and cytosolic ribosome (cluster 2: Rps9, Rps4x, Rps7), and two less connected clusters representing aminopeptidase activity (cluster 3: Npepps and Xpnpep1) and purine ribonucleotide binding (cluster 4: Adss, Gars, Tcp1, Hspe1, Acta2) (Figure 2). Furthermore, clusters 1 and 2 show medium confidence interactions with cluster 4 but not with each other.

Finally, KEGG pathway analysis was performed to characterize biological pathways in the differentially expressed proteins. The top pathways were chaperones and folding catalysis, peptidases and inhibitors, ribosomes, mRNA biogenesis and transporters.

## 4 DISCUSSION

The purpose of this study was to characterize and quantify early protein changes after MyD88 inhibition in order to gain insight into how blocking MyD88 signalling may lead to photoreceptor protection in the *rd10* mouse model of retinal degeneration. Using iTRAQ quantitative proteomic analysis, we demonstrated differential expression of 42 proteins in the treated retinas. A major class of proteins that was changed was crystallins, a family of proteins that function as stress response and anti‐apoptotic proteins. Additional chaperones and cell stress response proteins were also upregulated. This study is the first quantitative proteomics analysis of *rd10* mice treated with an experimental therapy and identifies a link between modulating inflammatory pathways and crystallin expression. These data suggest that inhibiting MyD88‐mediated signalling may enhance intrinsic tissue‐protective pathways.

### 4.1 Crystallins and other chaperone proteins

Our findings demonstrated that out of 20 upregulated proteins, seven belonged to the crystallin family. The α‐crystallins CryαA and CryαB were both significantly upregulated in the MyD88‐inhibited mice. The α‐crystallins are members of the small heat shock protein family and have molecular chaperone and anti‐apoptotic activities.^26^, ^29^ In addition to preventing cell death, studies using exogenous crystallins, crystallin core peptides or knockout mice demonstrated that crystallins suppress inflammation by acting on astrocytes and microglia.^30^, ^31^, ^32^ In the retina, intravenous delivery of recombinant α‐crystallin reduced retinal ganglion cell (RGC) death in a rat optic nerve crush model, which was associated with decreased microglia numbers and suppressed cytokine expression.^33^ A small peptide derived from αB‐crystallin reduced photoreceptor death in a mouse model of AMD^34^ and protected human retinal pigment epithelium (RPE) cells exposed to oxidative stress.^35^ Furthermore, in a uveitis mouse model, αA‐crystallin prevented inflammation and reduced retinal degeneration, whereas genetic loss of αA‐crystallin enhanced degeneration.^36^ Many studies have reported elevated αA and αB‐crystallin expression in neurodegenerations, including ocular hypertension, animal models of uveitis, diabetes and optic nerve transection^27^, ^37^ and AMD,^38^ and Alzheimer's disease and Parkinson's disease,^39^, ^40^ suggesting they play a common role in neurodegenerations as an intrinsic protective response to damage. In contrast, αA‐crystallins are detrimental in other disease conditions by promoting fibrosis, angiogenesis and proliferation of cancer cells,^29^ indicating cell‐specific and disease‐specific functions.

The βA1‐ and βA3‐crystallin proteins were also upregulated in MyD88‐inhibited retinas. Although less information is available about their role outside the lens, the β and γ crystallin superfamily share similarities to heat shock proteins, and increasing evidence supports their activity in neuronal remodelling and repair. Previous studies demonstrated that β‐crystallin and γ‐crystallin were increased in the retina and optic nerve after optic nerve crush, and delivery of βB2‐crystallin promoted axonal growth.^41^ Experiments in various animal models of retinal disease using overexpression of β or γ crystallins demonstrated their role in increasing survival of RPE,^42^ photoreceptors^43^ and RGCs^44^ and promoting RGC axonal growth.^45^ Overexpression of βB2‐crystallin also increased RGC survival after ON injury.^43^ βA3/A1 crystallin was highly upregulated in our study, and this protein is known to mediate lysosome function and autophagy in RPE,^46^ raising the possibility of a similar role in the *rd10* retina. Furthermore, βB2‐crystallin induced retinal CNTF,^45^ a well‐known neuroprotective molecule in the retina. Therefore, accumulating evidence suggests that β‐ and γ‐crystallins, delivered directly by overexpression or induced by MyD88 inhibition, are important mediators of retinal cell survival in animal models. Further studies will determine whether crystallins are required for photoreceptor protection after MyD88 inhibition, and whether enhancing chaperone and crystallin stress response activity provides sustained retinal protection in *rd10* mice.

Out of the 20 upregulated proteins, many are known to act as chaperones or to reduce cell stress. In addition to the seven crystallins already mentioned, we observed upregulation of chaperonin 10 and serine/threonine protein phosphatase PP1‐beta (Ppp1cbP), a nucleophosmin phosphatase that regulates cellular responses to genotoxic stress.^47^ Adenomatous polyposis coli protein 2 (APC2), which regulates the response to ER stress,^48^ and Uchl1, a neuroprotective ubiquitin carboxyl‐terminal hydrolase,^49^ also regulates oxidative stress.^50^ Chaperone proteins chaperonin 10 and Tcp1^51^ were also upregulated in the MyD88‐inhibited mice. These data suggest the possibility that the MyD88 inhibitor stimulates chaperone and crystallin expression, potentially as a tissue‐protective response.

To our knowledge, an effect of MyD88 signalling on crystallin expression has not previously been reported. Future work will determine whether induction of crystallins by MyD88 inhibition occurs in other tissues or is limited to retinal degeneration, whether the effect persists during the course of injury, and will determine the mechanism of upregulation. Interestingly, several studies demonstrated increased crystallin expression associated with neuroprotective agents in the retina. For example, long‐term photoreceptor protection by NT‐4 delivery from cell implants after sodium iodate‐induced retinal injury was also associated with increased β‐crystallins 3 months after injury, including several that we also detected (Crybb3, Cryba1 and Crygc).^52^ Metformin‐mediated photoreceptor protection was also associated with upregulated crystallin expression in the retina of *rd1* mice, an allelic strain to the *rd10* mouse used here, and αA‐crystallins were demonstrated to contribute to the therapeutic effect of metformin.^53^ Additionally, upregulation of αA‐ and αB‐crystallins and βA4‐ and βB2‐crystallins was associated with RGC survival induced by L. barbarum polysaccharide in a rat ocular hypertension model,^54^ further implicating the crystallins in therapeutic neuroprotection.

Expression of alpha, beta and gamma crystallins in RPE, neurons and glia within the retina have been reported in rodent and humans.^27^, ^28^ Unfortunately, attempts to determine the cell type(s) expressing the crystallins in *rd10* retinas using immunohistochemistry were not successful. Instead, we queried published single‐cell RNAseq data from wild‐type C57Bl/6 retina (https://singlecell.broadinstitute.org),^55^ and NMDA injected and light damaged mouse retinas (https://proteinpaint.stjude.org/F/2019.retina.scRNA.html).^56^ These databases showed that high expression levels of Cryab, Crybb3 and Crygs were detected in Muller glia and astrocytes, and lower levels of the other crystallins were detected in multiple cell types in the retina. All of the seven crystallins we identified in *rd10* were detected in the published single‐cell RNA analysis, showing agreement with our proteomics data.

Additionally, to address the possibility of non‐ocular sources of crystallins, we performed a query of single‐cell RNA sequencing data in the Protein Atlas database, which indicated that many of the crystallins are expressed outside the eye (http://www.proteinatlas.org). Furthermore, MyD88 is expressed in Muller glia as well as other cell types, including retinal microglia, bipolar cells and endothelial cells, http://www.proteinatlas.org. Therefore, it is possible that Muller glia and other retinal cells may respond to the inhibitor and are the source of crystallins, and crystallins may also have non‐ocular sources and enter the eye from the circulation.

The group of 22 downregulated proteins did not contain chaperone or stress response proteins. However, there was enrichment of ATPase and GTP‐binding proteins in the downregulated dataset. Also, cluster analysis indicated enrichment of proteins involved in peptide metabolism (Rps9, Eif4a2, Xpnpep1, Npepps) and pyrophosphatase activity (for example, Gnb1, Atp2b1, mt‐Atp6), suggesting changes in protein synthesis. It is currently unclear how these groups of proteins are related to MyD88 inhibition. Two photoreceptor proteins, rhodopsin and Gnb1, were also reduced which was unexpected due to the early timepoint prior to degeneration, but their decrease may be related to reduced protein synthesis proteins.

We did not detect changes in inflammatory proteins in the retinal samples at the timepoint tested despite previous studies from our group and others that showed cytokines and other inflammatory mediators are affected by blocking MyD88 signalling at the peak of degeneration. In fact, the proteomics analysis did not detect interleukins, Toll‐like receptors, chemokine ligand and receptors, NFkB, TNF or complement proteins in the high or medium confidence list. The absence of inflammatory gene expression may be because this early timepoint is prior to photoreceptor degeneration and its associated inflammatory changes. Therefore, MyD88 inhibition early in degeneration does not appear to block inflammation but may instead be stimulating intrinsic tissue stress responses through crystallins and other stress response proteins. Future studies using direct experimental validation will determine whether these proteins contribute to protection from MyD88 inhibition.

### 4.2 Limitations and future directions

Several limitations are noted. First, as mentioned above, 72 proteins that were identified by MS were not labelled with the isobaric tags and differential expression could not be assessed. However, this number represents only 1.2% of the total medium and high confidence proteins identified, which is unlikely to affect the overall conclusions of the study. Second, the study is focussed only on protein expression because post‐translational modifications in response to MyD88 signalling inhibition, such as phosphorylation or acetylation, could not be detected with our methods. Third, although iTRAQ is highly efficient and sensitive, standard confirmation analyses, such as Western blotting or ELISAs, would provide further validation. However, mass spectrometric sequencing of proteins provides high confidence identification compared to other methods that rely on antibody specificity. Finally, we deliberately chose an earlier timepoint to detect changes in the retina prior to extensive photoreceptor death, and future experiments would be performed to characterize expression of the differentially expressed proteins at additional timepoints to determine whether they show sustained expression changes.

## 5 CONCLUSIONS

This study presents the first iTRAQ analysis to quantify protein differences in degenerating retinas from mice treated with an experimental neuroprotectant molecule. In summary, *rd10* mice treated with the MyD88 inhibitor peptide showed a significant increase in chaperone and crystallins proteins and a decrease in peptide metabolism proteins. These findings indicate that neuroprotection by MyD88 inhibitor treatment is associated with induction of signalling pathways that reduce cellular stress and protein misfolding, suggesting enhancement of an intrinsic tissue‐protective response. Therefore, these findings identify candidate pathways that may contribute to neuroprotection in MyD88 inhibitor treated rd10 mice.

## CONFLICT OF INTEREST

The authors declare that they have no competing interests.

## AUTHOR CONTRIBUTION

**Tal Carmy‐Bennun:** Data curation (supporting); Investigation (supporting); Methodology (supporting). **Ciara Myer:** Data curation (supporting); Methodology (supporting). **Sanjoy K. Bhattacharya:** Funding acquisition (supporting); Methodology (supporting); Project administration (supporting); Resources (supporting); Software (supporting); Supervision (supporting); Writing‐review & editing (supporting). **Abigail S. Hackam:** Conceptualization (lead); Formal analysis (lead); Funding acquisition (lead); Investigation (lead); Project administration (lead); Supervision (lead); Visualization (lead); Writing‐original draft (lead); Writing‐review & editing (lead).

## ETHICAL APPROVAL

Approval for the use of the *rd10* mouse model in these experiments was obtained by the University of Miami IACUC committee.

## Supporting information

Table S1

## Data Availability

The data that supports the findings of this study are available in the manuscript and its supplementary material. Raw proteomics data have been deposited in the PRIDE (PRoteomics IDEntifications) database, Project accession: PXD024501, Project 10.6019/PXD024501.
