# Supplementary material for: Quantitative proteomic analysis after neuroprotective MyD88 inhibition in the retinal degeneration 10 mouse
Source: J Cell Mol Med. 2021 Sep 25;25(20):9533–42. doi: 10.1111/jcmm.16893 (PMC8505828; doi:10.1111/jcmm.16893)
Supplement: Supplementary file 1 — Table S1 [file JCMM-25-9533-s001.docx]

**Supplemental Table 1** Differentially expressed high and medium confidence proteins, with corresponding peptide number, percent coverage and MyD88 inhibitor/control ratios.
